# Developing a Parent-Focused Decision Aid to Promote Child-Inclusive Shared Decision-Making in Pediatric Oral Immunotherapy: Pragmatic Exploratory Feasibility Study

**DOI:** 10.2196/77782

**Published:** 2026-01-06

**Authors:** Junko Hayama, Kanako Yamamoto, Kota Hirai, Koichi Yamaguchi, Hiroyuki Mochizuki, Kazuhiro Nakayama

**Affiliations:** 1 Department of Nursing, School of Medicine, Tokai University, 143 Shimokasuya, Isehara, Kanagawa, 259-1193, Japan, 81 463 90 2084; 2 Graduate School of Nursing Science, St. Luke’s International University, Tokyo, Japan; 3 Department of Pediatrics, St. Marianna University School of Medicine, Kanagawa, Japan; 4 Department of Pediatrics, Tokai University School of Medicine, Kanagawa, Japan

**Keywords:** decision support techniques, shared decision-making, food hypersensitivity, oral immunotherapy, parents, child, family-centered care, quality-of-life

## Abstract

**Background:**

Shared decision-making is increasingly valued worldwide in pediatric care; nonetheless, its application in Japanese clinical practice remains in its early stages, particularly in areas with substantial medical uncertainty, such as food allergy (FA) management. Although oral immunotherapy is a promising option for children with FA, its long-term effectiveness and safety remain under evaluation, providing families with limited evidence to navigate emotionally complex decisions. Despite this clinical uncertainty, decision aids (DAs) are beneficial for organizing information and supporting patients and families in making value-congruent choices. Involving children in these decisions is increasingly recognized as ethically and developmentally appropriate. DAs clarify treatment options and promote informed collaborative decisions. However, most DAs target adult users and do not explicitly encourage engagement with children’s views.

**Objective:**

This study aimed to develop a culturally adapted DA for Japanese parents by considering their children’s preferences and perspectives.

**Methods:**

A paper-based DA was developed through iterative alpha testing and finalized by a multidisciplinary team. In total, 9 parents of children eligible for oral immunotherapy participated in this study and received the DA. Although intended for parents, the DA was structured to prompt reflection on the children’s involvement in decision-making. Parents completed structured questionnaires before and 1 week after receiving the DA to assess uncertainty, anxiety, and the burden of FA management. A total of 4 children completed the quality-of-life (QoL) questionnaire. Subsequently, all 9 parents and 4 children participated in semistructured interviews. Parents discussed how they used the DA, their perceptions of its clarity, and their interest in involving their children in decision-making. The children shared their thoughts about participating in decision-making.

**Results:**

All 9 parents read the DA and completed the follow-up assessment (100% retention rate). Among them, 4 children participated in pediatric QoL assessments and interviews. Parents’ Decisional Conflict Scale scores significantly decreased from 58.3 (SD 29.9) at baseline to 26.7 (SD 24.1) postintervention (*t*_8_=2.65; *P*=.03). The values clarity subscale also significantly declined, from 73.1 (SD 30.6) to 25.9 (SD 26.2) (*t*_8_=4.50; *P*=.002). No significant changes were observed in parental anxiety and QoL. Overall, 7 of the 9 parents explained the treatment options to their child, and 6 reported actively seeking their child’s feelings. The interview results suggested that the DA was associated with a shift in the family dynamic “from protecting to partnering.”

**Conclusions:**

Culturally adapted DAs appear practical and acceptable to Japanese families when making pediatric FA treatment choices. Facilitating parent-child dialogue may promote more inclusive decision-making. Nevertheless, further research with larger samples and longer follow-up periods is warranted to confirm these findings and refine the tool.

## Introduction

### Background

Pediatric food allergies (FA) affect approximately 8% of children worldwide and present ongoing medical and psychosocial challenges to patients and their families [1-3]. Oral immunotherapy (OIT) has emerged as a therapeutic option alongside traditional allergen avoidance and emergency preparedness; notably, it is gaining popularity in several countries, including Japan. Nevertheless, OIT involves considerable daily workload and prolonged commitment; moreover, it can provoke mild-to-moderate symptoms and, on rare occasions, anaphylaxis [1,4,5]. Thus, because multiple reasonable options exist and value concordance influences outcomes, OIT is a prototypical preference-sensitive decision. Therefore, these trade-offs should be evaluated systematically through shared decision-making (SDM), which aligns with the family’s values and risk tolerance. Additionally, the Canadian Society of Allergy and Clinical Immunology (CSACI) guidelines emphasize that SDM is ethically and clinically essential for OIT, ensuring that families make informed and personalized decisions [6].

SDM is a clinical practice model that integrates the best available evidence with patients’ and families’ values and can improve knowledge, reduce decisional conflict, and enhance adherence [7-9]. As a framework that supports the implementation of SDM, the Ottawa Decision-Support Framework (ODSF) identifies decisional needs, such as knowledge deficits, unclear values, and insufficient support, and organizes tailored interventions to address them [10,11].

Within the ODSF, patient decision aids (DAs) represent primary implementation vehicles, delivering evidence-based information, structuring value clarification, and prompting supportive dialogue. Recent systematic reviews have demonstrated that DAs are effective across diverse clinical contexts in increasing knowledge, promoting value-concordant choices, and reducing decisional conflict [10,11].

Implementing SDM in pediatrics entails additional complexity layers arising from a triadic structure, health care providers, caregivers, and the child, in which developmental stages, family roles, and emotional dynamics intersect [12,13].

Uncertainty regarding diagnosis, prognosis, and treatment outcomes constitutes a significant barrier to SDM in complex pediatric care; moreover, hierarchical power imbalances during clinical encounters further impede its implementation. Similarly, continuity of care, access to accurate and balanced information, and communication skills exert substantial influence. These patterns, synthesized in a recent scoping review of pediatric community health services, underscore the need for approaches supporting equitable partnerships and high-quality information exchange [14]. Furthermore, parents’ strong protective orientation may limit the elicitation and incorporation of children’s preferences. Thus, developmentally appropriate support for child participation and deliberately structured collaborative partnerships between parents and clinicians are essential [1,12,13]

In pediatric FA, anxiety regarding accidental exposure and ongoing at-home care workload imposes condition-specific emotional and practical burdens on families [1,6]. These condition-specific burdens intensify general barriers to pediatric SDM, making it necessary to design decision supports that not only structure information and value clarification but also surface and integrate the child’s developmentally appropriate perspective alongside parents’ values [14-16].

### Knowledge Gap and Study Aim

In Japan, pediatric OIT is not widely recommended in routine clinical practice, and many families rely primarily on allergen avoidance within tolerated ranges [5]. Nonetheless, domestic preliminary reports [4] have documented an increasing number of institutions offering pediatric OIT, currently exceeding 100 nationwide. Despite this growth, opportunities for families to view OIT as a realistic option and engage in SDM that incorporates their children’s preferences remain limited.

Importantly, these dynamics are more pronounced in the Japanese clinical context, where deference to medical authorities and high-context communication may amplify hierarchical power imbalances and hinder SDM implementation [17,18].

During emotionally charged visits, families may find it difficult to voice uncertainties, hopes, or questions [1,19]. Although children have the right to express their views on matters affecting them [16], meaningful participation in medical decisions remains limited. Moreover, Japan lacks OIT-specific DAs, and existing developments largely originate from outside Japan, leaving a gap in culturally adapted support. Consequently, there is a need for DAs that go beyond information provision and value clarification to activate dialogue, meet emotional needs, and enable parents to incorporate developmentally appropriate children’s views and feelings into their decisions. Therefore, this study aimed to develop and evaluate the feasibility and acceptability of a culturally adapted, parent-focused DA designed to facilitate child-inclusive dialogue in pediatric OIT settings in Japan.

## Methods

### Study Design

We conducted a pragmatic exploratory feasibility study to assess the newly developed DA for families eligible for pediatric OIT. This type of feasibility work commonly enrolls 10‐30 participants, which is an adequate range for identifying procedural issues and evaluating the initial signals of effect [20,21]. Guided by this benchmark, we enrolled 10 parents and 5 children. One parent-child dyad withdrew before the baseline assessment; therefore, the analyses included 9 parents and 4 children (N=13). Each participant completed structured questionnaires at baseline and 1-week postintervention, followed by a brief semistructured interview. To effectively integrate quantitative and qualitative data, numeric measures (eg, decisional conflict, state anxiety, and QoL) were paired with interview feedback (eg, parent-child communication and DA usability) [22,23]. This study was not powered to test efficacy but did generate preliminary data and highlight practical issues that should be addressed before a larger trial.

### DA Development

We developed a parent-focused booklet DA to support SDM by prompting parents to elicit and consider their child’s views and, where appropriate, to collaborate with the child, following a systematic development process [24], adhering to the Japanese adaptation of the International Patient DA Standards Instrument version 4.0, and meeting all 6 qualifying criteria [25].

Following the Ottawa Decision Guide, the DA is organized into 4 core sections: understanding the decision-making process, comparing treatment options, clarifying personal values, and assessing the current situation. To promote meaningful child involvement consistent with the UN Convention on the Rights of the Child [16], the DA includes brief information about the Convention and sample questions that parents can use to explore their child’s feelings and is formatted as an easy-to-use booklet for parents and children (Table 1).

**Table 1.** Contents of the Let’s Think Together About Treatment Options for Food Allergies decision aid.

Chapter	Contents	Setting
Option	Guidebook objectives Table of contents	Read
Step 1	Guidance on SDM^a^ How to make more informed decisions about treatment	Read
Step 2	Knowledge of illnesses and treatments available Choosing between elimination and OIT^b^ Understanding food allergies Understanding potential treatments and their characteristics Understanding the lifestyle and psychological impact of treatments	Read
Step 3	Value-based decision-making Clarifying what is important to you when you make a decision Colum: Children’s feelings about treatment Let’s ask your child about their feelings regarding the illness and treatment	Read Check Read Read and write
Step 4	Treatment options that are currently under consideration Clarifying your current feelings and organizing your concerns	Read Check

a SDM: shared decision-making.

b OIT: oral immunotherapy.

### Development Followed a User-Centered, Multistage Process

Formative interviews were conducted with 14 stakeholders, 5 parents, 3 children, and 6 health care providers, by purposively sampling families that had previously considered OIT (proceeded vs continued elimination; approval number 19R-272). Children expressed a desire to learn about options and to be invited to participate in OIT decision-making. Their input informed the parent-facing DA by adding nonleading prompts to elicit parents’ children’s views and by adopting age-appropriate wording.

Next, we synthesized evidence from the Japanese clinical guidelines [5], a systematic review of the impact of OIT on QoL, and a conceptual analysis of decision-making in pediatric chronic conditions. Using these inputs, a multidisciplinary panel (SDM specialists, pediatric allergists, and nursing researchers) specified the content, wording, and layout and produced a paper prototype.

Subsequently, the prototype was alpha tested with 10 parents of children with FA who had previously been considered for an OIT decision (approval number 21R-020). Acceptability was high: 9 (90%) and 1 (10%) rated the DA as “Excellent” and “Very good,” respectively. Parents judged the information to be balanced between elimination and OIT, and the feedback emphasized clearer headings, simplified language, and greater use of visuals. Revisions were made accordingly, and an improved DA was used here.

**Figure 1.**
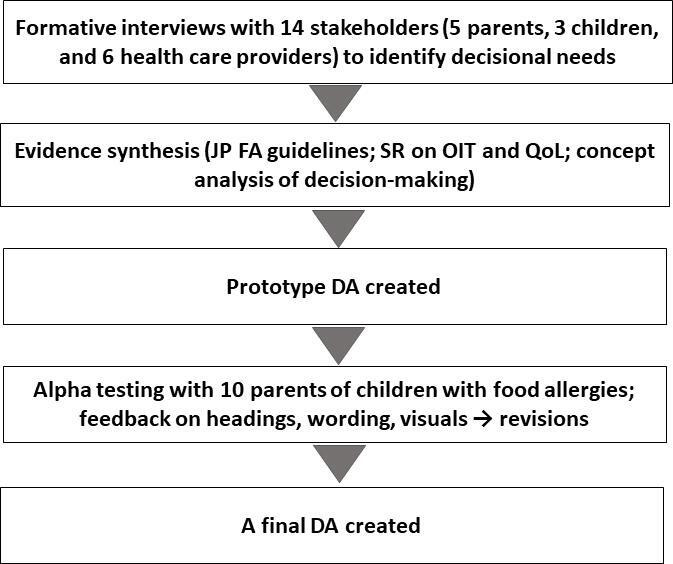
Flow diagram of the decision aid development process. DA: decision aid; FA: food allergy; JP: Japanese; OIT: oral immunotherapy; QoL: quality of life; SR: systematic review.

The development process is summarized in Figure 1, and the final DA is provided in Multimedia Appendix 1.

### Participants and Setting

The participants were recruited from a pediatric allergy outpatient clinic in Japan. Two groups of participants were eligible: parents and children. Parents could participate regardless of the child’s age, whereas children were eligible only if they were in grade 1 or higher in primary school (typically ≥6 y). We set the eligibility according to the treating physician’s clinical judgment regarding the appropriateness of discussing OIT in individual cases. In this study, “suitability for OIT” referred to children currently managed with an elimination diet for whom the physician judged that OIT could be considered and discussed as a potential option. Parent participation was not contingent on child participation; therefore, the parent and child sample sizes were not numerically matched.

The inclusion criteria are listed in Textbox 1.

Textbox 1. **Inclusion criteria.**

- Parents of children currently managed with an elimination diet for whom the treating physician judged that OIT could be considered and discussed.
- Children in grade 1 or above in a Japanese primary school (typically ≥6 y), with adequate cognitive capacity to participate in interviews and task-based procedures, and for whom the treating physician judged that OIT could be considered and discussed.

### Recruitment and Consent

Physicians and nurses conducted the study during clinic visits. We used two invitation pathways: parent-only and parent-child invitations. For parent-only invitations, the physician or nurse explained the study to the parent and, if interested, asked the parent to contact the research team via the email address or phone number listed on the information sheet to minimize any perception of coercion. For parent-child invitations, the physician or nurse explained participation separately to the parent and the child using age-tailored information sheets (lower elementary, upper elementary, and junior high versions). The parents then confirmed the willingness of the child. Enrollment proceeded only when both parents and children expressed interest, after which the parents contacted the research team via email or phone. Before any study procedures, all parents provided written informed consent, and the children provided age-appropriate assent.

### Intervention and Data Collection Procedure

#### Overview

We conducted this study between October 2022 and May 2023.

At the single participating clinic, we approached 10 parents and 5 children, and all agreed to participate. One parent-child dyad was excluded before baseline because OIT was initiated before questionnaire distribution; accordingly, this dyad was not included in the analytic sample. Data from 9 parents and 4 children who completed both the baseline and 1-week questionnaires and postintervention interviews were included in the analysis.

#### Baseline

After obtaining parental consent and child assent, the parents (9/9, 100%) and children (4/4, 100%) completed the baseline questionnaire.

#### DA Provided to Parents

At the next clinic appointment (2‐4 wk later), a physician provided a brief, nondirective orientation to the DA, highlighting that multiple treatment options existed and that the DA offered tips and prompts for SDM. To avoid influencing DA use or evaluation, we provided no option-specific counseling.

#### One Week Postintervention

At the 1-week follow-up, parents and children repeated the questionnaires and participated in brief semistructured interviews to explore their experiences with the DA.

### Quantitative Outcome Measures

**Figure 2.**
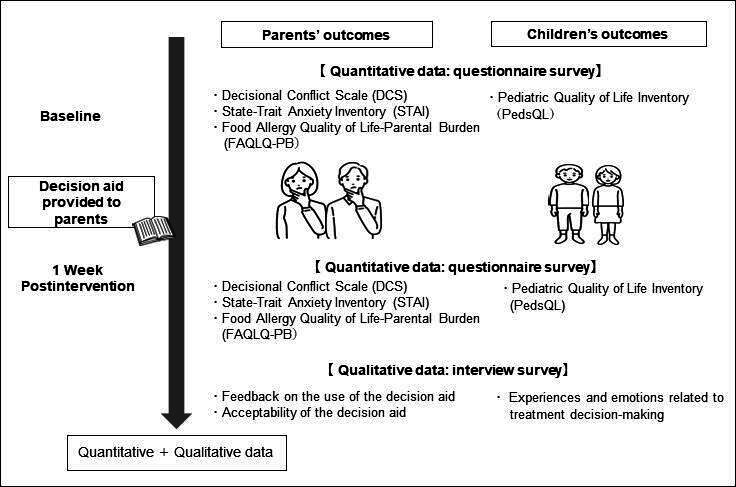
Study flow diagram of recruitment, decision aid intervention, and 1-week follow-up.

Guided by the ODSF, we prespecified the Decisional Conflict Scale (DCS) as the primary outcome and the State–Trait Anxiety Inventory, State Anxiety subscale (STAI-State), FA QoL–Parental Burden (FAQLQ-PB), and Pediatric QoL Inventory (PedsQL) as contextual measures, given evidence that DAs reduce DCS and improve decision quality [26,27]. We assessed parental outcomes, children’s health-related QoL, and postintervention feasibility and acceptability. We used validated Japanese versions with published reliability and construct validity for the DCS, STAI-State, FAQLQ-PB, and PedsQL. Self-administered questionnaires were completed at baseline and 1-week postintervention. We measured postintervention feasibility and acceptability using study-specific items, as displayed in Figure 2.

### Parental Outcomes (Pre- and Postintervention)

#### DCS, Japanese Version

This tool measures uncertainty and perceived difficulty in making health-related decisions [26]. The scale includes 5 subdomains: feeling informed, clarity of values, perceived support, uncertainty, and effectiveness of decision-making. Higher scores indicate greater decisional conflict. We chose this measure as the primary proximal outcome in the ODSF framework [10,27].

#### STAI, Japanese Version

This tool assesses the situational (state) components of anxiety [28]. We used only the state anxiety subscale in this study. Higher scores indicate increased anxiety. We included this measure to index the emotional burden relevant to preference-sensitive choices, consistent with the ODSF [27].

#### FAQLQ-PB, Japanese Version

This tool evaluates the psychological and practical burden among parents managing a child with a FA; higher scores indicate lower QoL [29,30]. To capture family-level impacts beyond decisional cognition, QoL sensitivity to SDM-oriented interventions has been reported in pediatric asthma trials [31].

### Child Outcomes (Pre- and Postintervention)

We used different versions of the Japanese version of PedsQL based on age groups (5‐7, 8‐12, and 13‐18 y) [32]. Higher scores reflect better QoL. We used it as a low-burden, developmentally appropriate child outcome (self-report when feasible; parent-proxy otherwise) and prioritized proximal parental outcomes.

### Parental Feasibility and Acceptability Items (Postintervention Only)

The following parental feasibility and acceptability items were used:

1. Two yes-or-no questions assessing parent-child engagement in decision-making: (1) “Did you explain the treatment options to your child?” and (2) “Did you ask your child how they felt about those options?”
2. Likert-type items on DA clarity, format, ease of understanding, perceived usefulness, and willingness to use similar aids in the future.
3. One open-ended question asked parents what additional information or support they would have found helpful.

### Statistical Analysis

We analyzed quantitative data using IBM SPSS Statistics (version 25; IBM Corp). Descriptive statistics were calculated for each outcome. We used paired-sample *t* tests (2-tailed) to compare pre- and postintervention DCS, STAI, and FAQLQ-PB scores. We set statistical significance at *P*<.05.

### Qualitative Data Collection and Analysis

#### Overview

One week following DA distribution, we conducted brief semistructured interviews with the participating parents (9/9, 100%) and children (4/4, 100%). All interviews were performed by the first author, a female registered nurse, academically trained in qualitative methods at the PhD level, with no prior relationships with participants or sites.

At the start of each interview, the participants were informed that the interviewer was an independent nursing researcher and university teacher with no prior relationship with them or the recruiting hospitals. We interviewed each participant once and recorded no field notes. After obtaining informed consent, we audio-recorded and transcribed all interviews. We provided the semistructured interview guides for parents and children in Multimedia Appendix 2.

#### Separateness and Modality

A trained interviewer conducted 9 individual parent interviews (5 in person, 4 online) and 4 child interviews (all in person). To maximize comfort, 1 child elected to be interviewed alone and 3 elected to be interviewed with a parent present.

#### Duration

Parent interviews lasted 20‐40 minutes; child interviews lasted 10‐30 minutes.

#### Data Management and Analysis

The transcripts were imported into NVivo 14 software (Lumivero) for data management. We analyzed the interviews using thematic analysis [33]. We analyzed parent interviews thematically using inductive semantic-level coding with iterative codebook refinement and peer debriefs. We summarized child interviews narratively (descriptive summaries with exemplar quotations) to contextualize parent themes and were not formally coded because of the small sample size in this feasibility study. We did not seek thematic saturation. We judged the analytical adequacy based on the coherence and stability of the parent themes and the illustrative value of the child narratives.

### Data Presentation and Interpretation

To facilitate comparison, we used a side-by-side joint display that aligned each quantitative outcome row with a single qualitative column (related category and, when informative, a de-identified exemplar quotation) and positioned conceptually similar themes in parallel across the 3 measures (DCS, STAI-State, and QoL). Side-by-side displays are widely used in mixed-methods health research to integrate quantitative results with qualitative evidence and support interpretation [22,34].

### Ethical Considerations

We conducted this study in accordance with the Declaration of Helsinki and the Ethical Guidelines for Medical Research Involving Human Subjects in Japan. The Ethics Review Board of our institution approved this study (approval numbers 22-AC044 and 22RC-040). All parents provided written informed consent and children provided age-appropriate assent. Interviews were audio-recorded with permission. We deidentified all data, removed potentially identifying information from transcripts and quotations, and stored files on password-protected systems accessible only to the research team. No compensation was provided.

## Results

### Parent-Child Characteristics

A total of 9 parents and 4 children participated in the study, and their characteristics are summarized in Table 2. Of the 9 parents, 8 were female. Their children, all deemed eligible for OIT by their physicians, exhibited a mean age of 7.6 (SD 4.2; range 2‐14) years and were predominantly male (6/9).

**Table 2.** Demographic and clinical characteristics of parent-child participants.

Characteristics	Participants
Parents (n=9)	
Age (years), mean (SD; range)	42.7 (5.7; 35‐52)
Sex, n (%)	
Male	1 (11.1)
Female	8 (88.9)
Occupation, n (%)	
Home worker	5 (55.6)
Part-time job	1 (11.1)
Self-employed	2 (22.2)
Other	1 (11.1)
Children’s age (years), mean (SD; range)	7.6 (4.2; 2-14)
Children’s sex, n (%)	
Male	6 (66.7)
Female	3 (33.3)
Children’s allergies (duplicate entries), n (%)	
Eggs	2 (22.2)
Peanuts	3 (33.3)
Other	7 (77.8)
History of anaphylactic shock, n (%)	
Yes	4 (44.4)
No	5 (55.6)
Children (n=4)	
Age (years), mean (SD; range)	10.75 (4.2; 7-14)
Sex, n (%)	
Male	3 (75)
Female	1 (25)
Children's allergies (duplicate entries), n (%)	
Walnut	3 (75)
Peanuts	2 (50)
Others	3 (75)
History of anaphylactic shock, n (%)	
Yes	2 (50)
No	2 (50)

Of these 9 families, 4 children who completed the postintervention QoL assessments and interviews comprised a nested subsample. These participating children had a mean age of 10.8 (SD 4.2; range 7‐14) years; 3 were male and 1 was female. They were allergic (with duplicate counts allowed) to walnuts (3/4, 75%), peanuts (2/4, 50%), and other foods (3/4, 75%). Two of the 4 patients had a prior history of anaphylactic reactions, whereas 2 had no such history.

### Quantitative Outcomes in Parents

Table 3 presents the detailed quantitative results. As the primary outcome, parental decisional conflict decreased from 58.3 (SD 29.9) at preintervention to 26.7 (SD 24.1) at 1 week postintervention, a mean reduction of 31.6 points (95% CI 4.09 to 59.11; *t*_8_=2.65; *P*=.03; paired *d*=0.88). Improvements were particularly pronounced in the subscales of values clarity (mean 73.1, SD 30.6 to mean 25.9, SD 26.2; *P*=.002), perceived support (mean 48.1, SD 32.8 to mean 20.4, SD 22.1; *P*=.04), and uncertainty (mean 62, SD 32 to mean 30.6, SD 23.2; *P*=.04).

**Table 3.** Parental outcome measures at baseline and postintervention following decision aid use.

Scores and subscales	Baseline, mean (SD)	1-week postintervention, mean (SD)	*t* test (*df*=8)	*P* value
DCS^a^				
Total	58.3 (29.9)	26.7 (24.1)	2.65	.03
Informed	54.6 (35.9)	25 (25)	1.89	.10
Value clarity	73.1 (30.6)	25.9 (26.2)	4.50	.002
Support	48.1 (32.8)	20.4 (22.1)	2.42	.04
Uncertainty	62 (32)	30.6 (23.2)	2.43	.04
Effective decision	54.9 (37.5)	30.6 (29.4)	1.59	.15
STAI^b^	37.9 (8.99)	35.9 (8.17)	2.03	.08
FAQLQ-PB^c^	25.6 (7.82)	26 (8.9)	−0.31	.77

a DCS: Decisional Conflict Scale.

b STAI: State–Trait Anxiety Inventory.

c FAQLQ-PB: food allergy QoL, parental burden.

For secondary outcomes, STAI-State decreased from 37.9 (SD 9) at preintervention to 35.9 (SD 8.2) at 1 week postintervention; the mean difference was 2 points (95% CI −0.27 to 4.27; *t*_8_=2.03; *P*=.08), corresponding to a moderate standardized effect (paired *d*=0.68). FAQLQ-PB exhibited little change (mean 25.6, SD 7.8 to mean 26, SD 8.9); the mean difference was −0.4 points (95% CI −4.29 to 3.54; *t*_8_=−0.31; *P*=.77), with a negligible standardized effect (paired *d*=−0.08).

### Qualitative Explanations of Parental Outcome

#### Overview

Overall, 3 primary themes emerged from the interview data (Textbox 2). The first concerned communication with physicians, including environmental constraints and hesitation to voice concerns. The second pertained to emotional reactions and difficulties processing information related to treatment decisions. The third reflected emotional burdens, such as anxiety and uncertainty, which parents described before using the DA.

Textbox 2. **Key category and illustrative quotations.**

**Difficulties in physician communication**

- Time constraints: “Clinic is crowded; I hesitate to ask.”
- Need guidance: “When my child wants to eat, I’d like direction.”
- Uncertainty about asking: “I’m never sure how much I can consult.”

**Challenges in obtaining reliable information**

- Conflicting information: “Online advice is contradictory and confusing.”
- Information overload: “There’s so much data it’s overwhelming.”
- Child’s desire for autonomy: “I want to eat safely and have a say.”

**Difficulties in coping with emotional uncertainty**

- “I’m still anxious because there’s so much I don’t know.”

#### Difficulties in Physician Communication

These include difficulties related to interactions with physicians and obtaining reliable information. Within the first theme, parents described feeling constrained by busy clinical environments and were uncertain whether it was appropriate to voice their concerns. This was illustrated by comments such as “In outpatient settings, there are usually many people, so I felt it wouldn’t be right to take up too much time just for myself.”

#### Challenges in Obtaining Reliable Information

Under the second theme, parents reported frustration with conflicting or overwhelming online resources, for example, “Sometimes completely contradictory information comes up, right? I look things up because I don’t understand, but it just ends up confusing me even more” and “When I search the Internet, of course, information comes up. However, when I open it myself, there’s so much information that it becomes overwhelming.” These barriers appeared to directly contribute to high levels of decisional conflict before DA use.

#### Difficulties in Coping With Emotional Uncertainty

Several parents described feeling anxious or overwhelmed when considering treatment options, particularly because of uncertainty and lack of prior knowledge. Expressions of worry, such as “I’m still anxious because there’s so much I don’t know,” highlighted the emotional strain experienced before using the DA. Emotional stress often coexisted with difficulties in processing information and hesitancy about how to proceed.

### DA Acceptability and Parent-Child Communication

Parental responses regarding the acceptability of DA and parent-child communication are summarized in Table 4. All 9 parents (9/9, 100%) reported having read the DA, supporting its feasibility for home use. Most parents responded positively when asked about its acceptability: 89% (8/9) answered “yes” or “somewhat yes” to “was the DA easy to understand?” Nevertheless, 33% (3/9) reported writing in the open-ended sections of the DA. Although this may limit engagement with the writing component, it does not reflect poor acceptability. Rather, parents explained that because an OIT decision was not imminent, they did not feel the need to record their thoughts at that time. Instead, DAs are primarily used as reading tools or discussion guides in family conversations.

**Table 4.** Parental responses on collaboration with children, decision aid acceptability, and additional needs with illustrative quotations.

Variables	Quantitative (n=9), n (%)	Key category and illustrative quotations
Collaborate		
Explained options to the child	Fully, 5 (56) Partial, 2 (22) Limited, 1 (11) None, 1 (11)	Assessing understanding: “I realized my child was actually thinking about the treatment...”
Considered child’s feelings	Definitely, 4 (44) Somewhat, 2 (22) Neutral, 1 (11) Not, 2 (22)	Respecting feelings: “You can’t move forward without asking the child first.”
Acceptability		
Read the DA^a^	Yes, 9 (100) No, 0 (0)	Guide usefulness: “Reading the guide made the steps clear to me.”
Wrote in the DA	No, 6 (67) Yes, 3 (33)	Family reflection: “It’s good to take this home and think about it together.”
DA clarity	Definitely, 5 (56) Somewhat, 3 (33) Neutral, 1 (11) Not very clear, 0 (0) Not clear, 0 (0)	Reassurance: “Written explanations gave me peace of mind and were incredibly useful.”
Additional needs	—^b^	Early information: “I would have liked to receive information about OIT right after the diagnosis.” Peer stories: “Hearing others’ experiences would be helpful as a reference.”

a DA: decision aid.

b Not applicable.

Additionally, DA facilitated parent-child communication. When asked, “Did you explain the treatment options to your child?” 78% (7/9) of the parents responded affirmatively. Moreover, 66% (6/9) said they “listened to their child’s feelings about those options.” Parents emphasized that the DA prompted them to consider and discuss their child’s perspective and values in greater depth than before.

Furthermore, qualitative responses revealed that although some parents were reluctant to write in the DA, they found structured prompts helpful in organizing family discussions.

### Parent-Child Collaboration and Pediatric QoL

Four parent-child dyads participated in this study. Table 5 presents each dyad’s decision-making collaboration, along with the child’s PedsQL scores and illustrative quotations.

**Table 5.** Child engagement in decision-making processes and Pediatric Health-related Quality of Life; scores at baseline and 1 week postintervention following decision aid use.

Dyad (years)	Parent-reported items	PedsQL^a^^b^ (child), baseline 1-week	Child voice	Decisional conflict	Preference direction	Child quotation
A (7 y)	Explained options: yes Asked feelings: somewhat agree	93.5, 93.7	Minimal (“mother decides”)	Not expressed or low	Elimination	“My mother decides… I don’t know.”
B (10 y)	Explained options: yes Asked feelings: agree	97.8, 94.6	Clear (asks for dialogue)	Mild–moderate	Interested in OIT^c^	“I want to talk more… I’ve been kind of thinking about it.”
C (14 y)	Explained options: yes Asked feelings: agree	97.8, 96.7	Clear (requests participation)	Low (direction set)	Favoring-OIT	“I’d like to try OIT and have a say when deciding.”
D (14 y)	Explained options: yes Asked feelings: somewhat agree	92.4, 96.7	Clear (reasoned avoidance)	Low (stable stance)	Elimination	“I’m fine to keep elimination; I don’t need treatment talks.”

a PedsQL: pediatric health-related Quality of Life.

b Note: PedsQL indicates pediatric health-related QoL; higher scores reflect better HRQoL.

c OIT: oral immunotherapy.

All 4 parents (100%) reported explaining the treatment options to their children, and 3 of the 4 (75%) further stated that they asked how their children felt about those options. The PedsQL scores were uniformly high at preintervention (range 92.4‐97.8) and demonstrated minimal change postintervention (range 92.4‐96.7).

Child involvement varied by age. In lower elementary school (7 y), expressions of agency were minimal, and no verbalized conflict was noted (dyad A: “My mother decides… I don’t know.”). In upper elementary school (10 y), preferences were emerging yet ambivalent, with mild-to-moderate conflict (dyad B: “I want to talk more… I’ve been kind of thinking about it.”). In junior high school (14 y), positions were clearer and conflict was low, but directions diverged, favoring OIT (dyad C) over continued elimination (dyad D). For example, the 7-year in dyad A stated, “My mother decides,” whereas the 14-year in dyad C, after reading the DA with a parent, wished “to have a say.” Multimedia Appendix 3 presents de-identified excerpts from the separate parent and child interviews, integrated and organized by dyad (A–D).

## Discussion

### Principal Findings

This feasibility study examined a culturally adapted parent-focused DA to support SDM for families considering pediatric OIT in Japan. Quantitative results indicated reduced parental decisional conflict, and interviews suggested greater engagement in parent-child dialogue and heightened awareness of children’s involvement in decisions. Collectively, these findings provide preliminary support for integrating SDM tools into pediatric allergy care.

### Reduction in Parental Decisional Conflict

The DA used in this study appeared to support a reduction in parental decision-making conflicts, particularly in the DCS subscales of value clarity, perceived support, and uncertainty. These results are consistent with those of previous trials on OIT-specific DAs [35]. Additionally, our study provides descriptive within-participant change data from clearly defined baseline measurements, suggesting that DA helps parents better understand treatment options and reflect on personal values in the context of complex, preference-sensitive decisions. When there was no single “correct” choice, the DA structure, which encourages clarification and comparison, may have contributed meaningfully to reducing decisional stress. Notably, changes observed over a 1-week period may reflect influences beyond DA exposure. Additional clinician contact or family discussions can improve perceived support and value clarity, whereas allergy-related events or conflicting online information can increase uncertainty and anxiety. These measurement effects may also have contributed. Interviews documented parent-child dialogue after DA receipt, consistent with gains in value clarity and support; however, because co-interventions and information seeking were not systematically recorded, observed DCS reductions should be interpreted as preliminary and potentially contingent on unmeasured influences. Conversely, emotional outcomes such as anxiety and QoL did not demonstrate any considerable changes. This divergence between the cognitive and emotional domains has also been noted previously, suggesting that reducing uncertainty does not always lead to immediate emotional relief, particularly in high-stakes decisions such as OIT, where safety concerns persist [3,19].

### Emotional Outcomes and Narrative Support

To support an emotionally responsive SDM model, DAs should address factual content and users’ emotional needs. Recently, a narrative review noted that parents contemplating OIT can be confused by inconsistent or nonevidence-based online information and often report anxiety about allergic reactions, highlighting the importance of SDM approaches and DAs that help address misinformation while acknowledging parental concerns. This is particularly crucial, as parental fear and anxiety are recognized globally as major factors influencing treatment decisions for chronic conditions such as FA [1-3]. Similarly, our qualitative theme of “difficulties in coping with emotional uncertainty” echoes that observation and underscores the importance of embedding affective support, such as patient stories or peer testimonials, within future iterations of the DA. Nonetheless, the lack of considerable changes in anxiety and QoL may reflect the limited short-term sensitivity of the scales used and the brief 1-week observation period. The used QoL measure, which focused on chronic allergy management, may have been insufficiently sensitive to capture short-term shifts resulting from the decision-making experience. Additionally, continued parental expressions of concern in the interviews suggested the persistence of underlying uncertainty about OIT and its long-term effects, even after DA use. Collectively, these findings highlight the need for DAs to include information and components offering emotional support, reassurance, and peer feedback. As emphasized in previous studies, SDM is both a cognitive and relational process that requires supportive communication and trust [9,10]. Narrative elements, such as stories from similar patients, can promote reflection, empathy, and engagement, particularly in emotionally taxing contexts such as OIT [9,36,37]. Although the DA emphasizes evidence-based content and parent-child dialogue, future versions may benefit from incorporating narratives or links to peer support to foster emotional reassurance and decision confidence [38].

### Timing and Multifaceted DA Use

All the parents read and rated the DA positively, indicating their initial engagement. Nevertheless, the intensity and mode of use varied, underscoring that the parent-focused DA served multiple functions, information, dialogue prompts, and planning, rather than a single point-of-decision tool. Notably, many families continued to avoid allergens during the study and were not immediately pressured to initiate OIT. This context may have reduced the perceived need for written reflections or explicit decision-making.

Crucially, 1 parent expressed a desire for earlier intervention, stating, “I would have liked to receive information about OIT right after the diagnosis.” Furthermore, our needs assessment revealed that families often seek clarity on the flexibility of OIT, specifically whether “treatment can be stopped and later restarted or tried again” after a period of discontinuation. These themes point to an anticipatory role for the DA, positioning it early to support understanding and planning, and not only immediate choice.

FA is a chronic condition that requires ongoing management, and treatment decisions may need to be reassessed as children grow and their lifestyles change [1,2,6]. For instance, starting daycare or school, increased exposure to shared meals, and a child’s curiosity or desire for autonomy may prompt families to reconsider OIT, even after extended periods of allergen avoidance. Accordingly, families benefit from clear information about the revisitable and stepwise nature of OIT decisions, including the option to defer, pause, or reconsider treatment, so that the choice is not experienced as irreversible [1,2,6]. In pediatrics, decisions warrant periodic reappraisal as children mature and assume greater autonomy [15,39].

Prior work indicates that DAs are valuable for decision-making and reflective preparation. DAs can help patients clarify their values and enhance their readiness to make future choices. Additionally, they support preference articulation before clinical encounters [40], foster emotional engagement and reflective thinking through the inclusion of narratives [36], and allow patients to organize their concerns and values before consultations [19]. Future research should explore when and for whom such DAs should be offered to maximize their effects.

### Facilitation of Parent-Child Collaboration

This feasibility study suggests that the DA serves not only as an informational tool but also as a prompt for parent-child dialogue, making children’s perspectives and preferences, which are infrequently elicited during clinical encounters, more visible [1,13,41]. Several parents reported that they had not previously asked their child about treatment preferences, and some recognized clear opinions for the first time when using the DA. Concurrently, certain parental statements appeared to invite child compliance or deferral, potentially diminishing the child’s own voice [14,41]. Conversely, when parents asked nonleading decision-related questions, children tended to offer fuller accounts, consistent with reports that interaction styles influence children’s participation [13,15,39].

The observed pattern was consistent with developmental theory. In early school-age years, self-expression is limited; in upper elementary years, interest and nascent preferences emerge but often remain ambivalent; and in adolescence, positions become more autonomous and self-referential [1,15,42]. Aligning with this gradient, some children prefer to participate directly in decisions, whereas others choose to defer to their parents, underscoring the need for developmentally aligned and flexible support for SDM [13,43]. To support this flexibility, future iterations of the DA should incorporate explicit parent-facing guidance on strategies for child engagement tailored to different developmental stages. These findings are also concordant with those of reviews emphasizing both the importance of children’s involvement in medical decisions and the heterogeneity in how that involvement is expressed [13,41].

Overall, parents can use DA to elicit and incorporate their children’s views into clinical decision-making, and age-aligned design features are likely to be useful [12,15,39,42,43]. To translate these insights into practice, the proposed DA revisions add explicit, developmentally tailored parent guidance: meaning-making with concrete examples and illustrations in early school age; value prioritization by listing 2 to 3 advantages and concerns in upper elementary school; and self-referential reasoning combined with family consensus in adolescence. These staged supports enable children to select their preferred level of involvement and make triadic child-parent-clinician collaboration easier to implement. To build an evidence base, subsequent studies should evaluate age-stratified DA versions in larger, more diverse samples and co-design protocols for triadic collaboration that include parent-facing guidance [1,6].

### Implementation in Japanese Clinical Settings

The DA includes prompts designed to help parents clarify their values and encourage dialogue with health care providers. However, several parents reported being unsure how to use the DA or feeling reluctant to ask questions because physicians were busy. Therefore, the effectiveness of DAs depends on both the content and the context in which they are introduced. Cultural norms in Japan, such as respect for medical authorities and hesitation to speak up, may inhibit families from actively engaging with decision-support tools, particularly during time-pressured consultations.

Unlike our Japanese setting, where deference to medical authorities and time pressure can dampen questioning, North American programs have actively evaluated and implemented OIT-focused, formal SDM workflows. A recent study reported a pediatric product-agnostic OIT DA with high acceptability and low decisional conflict among caregivers, positioning DAs as a practical adjunct to clinical encounters [35]. Consistent with this, national CSACI guidance [6] explicitly promotes patient-oriented, preference-sensitive OIT decisions, shared responsibility between families and the health system, and organizational solutions that embed SDM tools beyond the physician-patient dyad (eg, team-based introduction and previsit use). Conceptually, SDM in FA also emphasizes that clinicians must understand “where the patient is coming from,” with DAs assisting values clarification rather than replacing dialogue, an approach that aligns with our qualitative finding that parents used the DA to “pause and reflect” even when consultation time was limited [1]. Taken together, these comparisons suggest that the previsit distribution and nonphysician-led onboarding of DAs are likely to mitigate Japan-specific barriers and facilitate shared deliberation within routine care.

Building on this mechanism, programs should specify when and for whom a DA is most useful. Likely triggers include decisional uncertainty, divergent family preferences, and limited consultation time. Clinicians can use brief, neutral cues to normalize values clarification without extending visits. Clear signposting on how to use the DA, age-appropriate sections for children, and plain-language summaries can further lower barriers where questioning is difficult.

Parents valued the credibility of the DA, noting that its development by health care professionals increased their trust in and willingness to use it. Additionally, receiving a DA allowed them to pause and reflect, suggesting that DAs can offer a psychological space that is not always available in typical consultations. The qualitative findings suggest that the DA served as a valuable reflective tool because the cultural context inhibited open questioning during consultations. Integrating SDM tools into routine care workflows, with endorsement from medical staff, can bridge cultural barriers and promote shared deliberation.

### Implications for Health Care Practice and Policy

Mechanistically, the DA structured and balanced information helps organize concerns and form realistic expectations, thereby potentially reducing uncertainty. Nonleading decision-related questions from parents promote clarity of values and shared understanding within family conversations. Furthermore, developmentally aligned, parent-facing guidance can enable children to choose their preferred level of involvement and may facilitate triadic child–parent–clinician collaboration. These preliminary findings offer cautious insights into enhancing SDM in pediatric allergy care through improved health care practices and supportive policies.

Therefore, tailored strategies are crucial. Considering that health care organizations and their configurations vary considerably across countries, effective SDM integration warrants tailored workflow solutions [13]. The potential role of nurses in this context is particularly noteworthy. Nonphysician health care professionals, including nurses or educators, can efficiently introduce and support DA use, particularly in time-limited outpatient settings. Therefore, distributing the DA before clinical encounters or having nonphysician professionals, such as nurses or certified allergy educators, briefly introduce the tool may be more practical. Nurses, particularly certified allergy educators in Japan, may be well-positioned to facilitate DA use by initiating conversations, clarifying treatment options, and supporting family communication.

Furthermore, DA integration outside the consultation room is essential. Additionally, providing a digital version accessible at home or in waiting areas may help families engage with the content at their own pace, as previous studies have emphasized that DAs are more likely to be used outside consultations. Institutional measures, including SDM training and supportive care protocols, may gradually increase the clinical environment’s readiness to incorporate decision-support tools. Furthermore, the successful integration of DAs and SDM principles into routine care will likely require a collaborative, multidisciplinary approach involving physicians, allied health professionals, and dedicated nursing staff. These directions are consistent with international recommendations, such as those of the CSACI, emphasizing the value of SDM in allergy care [6].

Beyond such system-level measures, the chronic and recurrent nature of FA itself underscores the need to prioritize the “knowledge” function of DAs. FA care entails recurrent choices across the child’s life course, as developmental transitions (eg, entry to daycare or school, expanding peer eating contexts, and emerging autonomy) prompt families to revisit OIT even after prolonged avoidance. Acquiring accurate knowledge in advance is not only a proximal outcome of DA exposure but also an anticipatory resource for future deliberation. Consistent with the International Patient DA Standards, knowledge is a core component of decision quality [44]. Notably, a Cochrane review demonstrated that patient DAs consistently improve knowledge and the accuracy of risk perceptions and can be used before consultations without harming satisfaction or health outcomes [11]. Thus, incorporating a brief DA-aligned knowledge assessment would strengthen our evaluation and clarify the pathway by which an evidence-focused DA may reduce decisional conflict in the short term while supporting repeat decisions over time. Moreover, recent FA guidelines underscore structured education and consent as prerequisites for OIT preparation, reinforcing the rationale for early preparatory DA use to scaffold later reevaluations as circumstances change [6].

### Limitations and Future Research Directions

This single-center feasibility study was limited by its small sample size. A post hoc sensitivity power analysis indicated that, at 2-sided *α*=.05 with 80% power, the minimal detectable standardized effect size was approximately 1.06 for n=9, indicating that only very large effects were detectable. While the primary outcomes (DCS) improved, estimates for the secondary outcomes (STAI and FAQLQ-PB) were imprecise; therefore, confirmation with adequately powered samples is warranted.

The participating children spanned a wide age range, and the analyses did not prespecify age stratification, rendering the observed age-related patterns descriptive rather than inferential. The 1-week follow-up was likely insufficient to capture the psychosocial effects. Coronavirus disease 2019–related constraints limited face-to-face interactions and may have influenced the recruitment and interview procedures. Finally, knowledge acquisition, which is an important target of DAs, was not measured. Future work should enroll larger and more diverse samples, including prespecified age strata, and extend the follow-up period to evaluate age-specific outcomes and engagement. Comparative qualitative approaches and case studies (eg, contrasting subgroups by age, disease severity, or parent-child dyad dynamics) may elucidate the context-dependent use of decision support. Iterative refinement of the parent-focused DA, together with co-designed, age-appropriate child components or digital modules, could improve process quality and enable the longitudinal assessment of knowledge gains.

### Conclusions

A culturally adapted DA may mitigate parental decisional conflict and improve parent-child communication in Japanese pediatric FA care. Despite the small-scale setting, these findings lay the groundwork for larger studies and the future implementation of DAs in routine pediatric care. Future research should focus on co-design approaches, long-term outcome evaluations, and the integration of DAs into multidisciplinary care frameworks to support family-centered and value-based decision-making in allergy treatment. Future work should involve large multisite trials to confirm its effectiveness, coupled with family co-design and age-specific adaptations.

## Supplementary material

10.2196/77782 Multimedia Appendix 1 English translation of the decision aid.

10.2196/77782 Multimedia Appendix 2 Semistructured interview guides (parent-child).

10.2196/77782 Multimedia Appendix 3 Deidentified parent-child excerpts by dyads A-D.

## Abbreviations

CSACI: Canadian Society of Allergy and Clinical Immunology
DA: decision aid
DCS: Decisional Conflict Scale
FA: food allergy
FAQLQ-PB: Food Allergy Quality-of-Life Questionnaire–Parental Burden
ODSF: Ottawa Decision-Support Framework
OIT: oral immunotherapy
PedsQL: Pediatric Quality-of-Life Inventory
QoL: quality-of-life
SDM: shared decision-making
STAI: State–Trait Anxiety Inventory
