# Supplementary material for: Developing a Parent-Focused Decision Aid to Promote Child-Inclusive Shared Decision-Making in Pediatric Oral Immunotherapy: Pragmatic Exploratory Feasibility Study
Source: J Particip Med. 2026 Jan 6;18:e77782. doi: 10.2196/77782 (PMC12774402; doi:10.2196/77782)
Supplement: Multimedia Appendix 1 [file jopm-v18-e77782-s001.pdf]

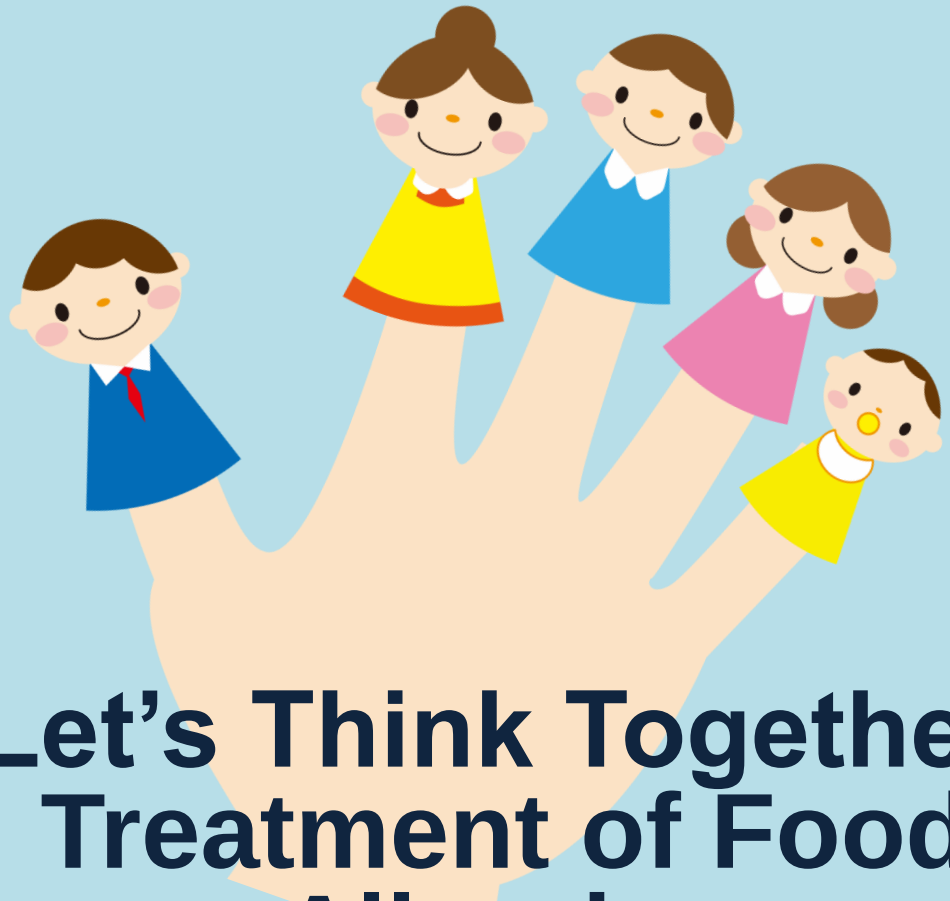

Let’s Think Together. Treatment of Food Allergies

—To all those who are considering treatment options—

This guidebook is designed to assist those considering food allergy treatments. It helps in obtaining information on the treatments currently available and in making more informed decisions.
We would like to encourage you to learn more about food allergy treatments, and to make more informed decisions by discussing matters with your healthcare provider, your child, and your partner.

Step 1

How to make more informed decisions about treatment

Those of you who have received this guidebook are probably concerned about their child's food allergy.
A food allergy is known to be a condition that can greatly affect the quality of life of a child and their parents. This is because eating, which is an essential part of the child's daily routine, can be life-threatening.

Parents may be particularly concerned about the treatment of their child's food allergy. In Japan, elimination diet therapy and oral immunotherapy are two treatments currently in use.

This guidebook is intended to support more informed decisions regarding treatment options.

A more informed decision means that the child and their family are fully informed about their treatment, consider what is important to them when making a decision, and decide on a treatment with a better understanding of their situation. By feeling satisfied about the decision, parents and children can expect to fully adhere to the treatment plan, which in turn will make it more effective.

This guidebook is designed to assist you in better understanding food allergies and to help you make more informed decisions about treatment options. We hope it will be helpful to you.

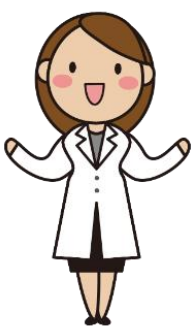

Step 1

How to make more informed decisions about treatment

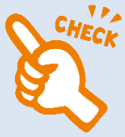

This guide is suitable for you if:

- Your child has been diagnosed with a food allergy and you would like to obtain information about treatment options.
- You are currently eliminating the allergenic food, but would like to consider more advanced treatment in the future.
- You are considering oral immunotherapy and want to compare the difference between this and elimination diet therapy.
- You wish to clarify your thoughts on treatment and discuss them further with your healthcare provider and your child.

☐ **This guidebook is not intended to oblige you to make a decision about treatment.**

Rather, it is designed to help you organize your feelings about your child's present food allergy treatment and to assist you in discussing this with your physician and other healthcare staff. Even after you have made a decision, you can change your mind at a later date. If you have any questions, please feel free to contact us at any time.

Some of you may prefer your physician to decide the method of treatment. Even in such cases, you may want to use this guidebook to check details of the treatment.

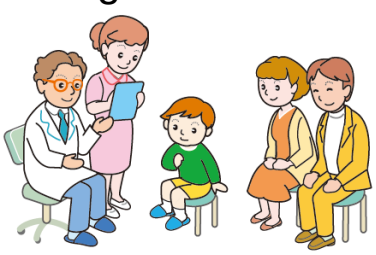

This guidebook is designed to help you organize your thoughts while reading through the following steps. Please follow these steps when you read it for the first time.

Follow the five steps

Step 1 How to make more informed decisions about treatment

Step 2 Understanding food allergies
Understanding potential treatments and their characteristics

Step 3 Understanding the lifestyle and psychological impact of treatments

Step 4 Clarifying what is important to you when you make a decision

Step 5 Clarifying your current feelings and organizing your concerns

Step 2

Understanding food allergies

In **Step 2**, we will identify the correct knowledge about food allergies.

What kind of illness is a food allergy?

A food allergy is a condition in which your body perceives a particular food as a threat (sensitization), which in turn causes various symptoms (allergy symptoms).

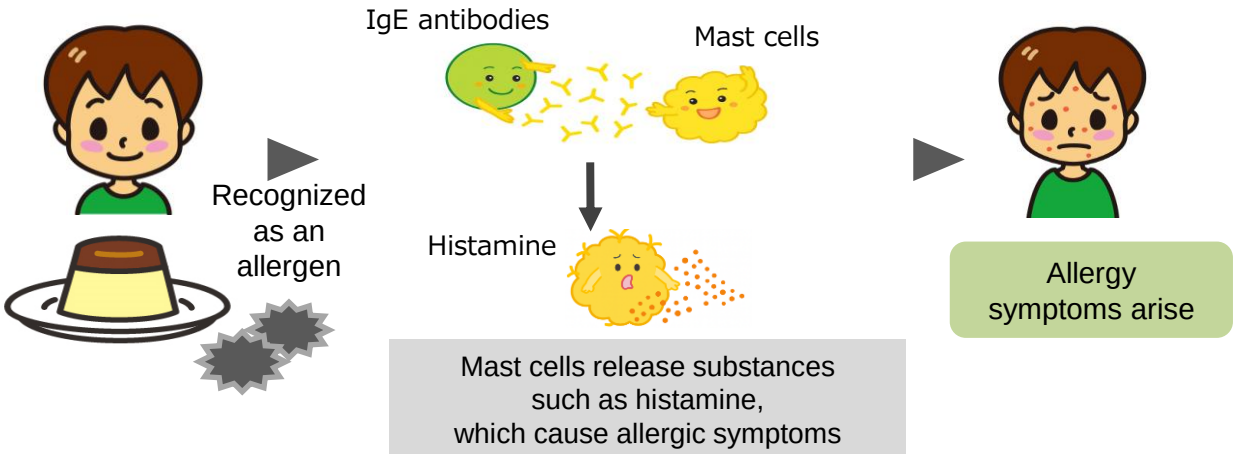

What kinds of foods cause food allergies?

All foods (proteins) have the potential to cause allergies. Egg, milk, and wheat allergies are common in preschool children, while fruit, buckwheat (in Japanese, *soba*), and shrimp allergies are common from elementary school age onward. People tend to judge for themselves which foods are causing the allergy, but allergies **are properly diagnosed by a physician** based on the history of a child's allergy symptoms, the results of blood tests and sensitivity tests, and so on.

0 years old		1 year old		2-3 years old		4-6 years old		7-19 years old	
Egg	62%	Egg	45%	Egg	30%	Egg	23%	Other	24%
Milk	20%	Other	20%	Other	24%	Other	23%	Crustaceans	16%
Other	11%	Milk	16%	Milk	20%	Milk	19%	Egg	15%
Wheat	7%	Fish Roe	7%	Wheat	8%	Crustaceans	9%	Buckwheat	11%
		Wheat	7%	Buckwheat	8%	Fruits	9%	Wheat	10%
				Peanut	5%	Peanut	6%	Fruits	9%
				Fruits	5%	Buckwheat	6%	Milk	8%
						Wheat	5%	Fish	7%

What kinds of symptoms are caused by food allergies?

Various symptoms are caused by food allergies, such as itchiness, rashes, stomachache, and shortness of breath, and these are dependent on a child's physiological characteristics, their physical condition at the time, and other factors.
Furthermore, it is necessary to be sufficiently aware that some symptoms such as anaphylactic shocks* may be life-threatening.

It is still not fully understood what causes the human body to perceive a particular food as a threat (sensitization). At present, it is thought that an abnormal immune response is brought on by sensitization from the skin, mucous membrane, and so on, or sensitization via the respiratory tract.

Key symptoms

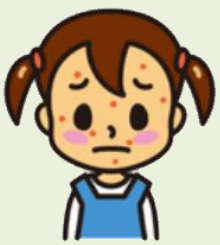

Symptoms of the skin, eyes, and mucous membrane

- Itchiness/rash
- Red eye
- Swollen eyelids
- Discomfort/swelling in the mouth and throat

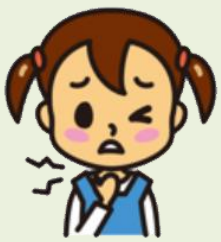

Symptoms of the respiratory system

- Runny nose
- Hoarse voice
- Coughing similar to a dog's bark
- Feeling of constriction in the throat
- Coughing, shortness of breath
- Puffing and wheezing

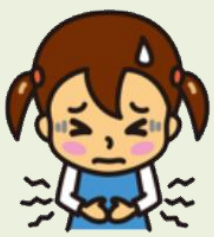

Symptoms of the digestive system

- Stomachache
- Nausea
- Vomiting
- Diarrhea

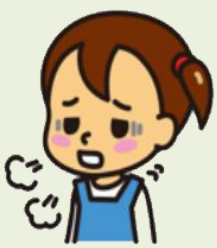

Symptoms of the nerves/circulatory system

- Faster pulse rate (tachycardia)
- Irregular pulse rate
- Cold hands and feet
- Pale lips and fingernails
- Lack of energy, bad temper
- Tiredness

*Symptoms vary depending on the individual.
*Anaphylaxis refers to more intense allergic symptoms occurring in the whole body, while anaphylactic shock refers to a state of shock caused by low blood pressure, etc.

Do food allergies get better over time?

It is said that many food allergies that appear during infancy get better as the child grows up.
There have been various reports, but for example**, reports say that 50% of children acquire a tolerance* to egg allergies by the time they are four years old**, **with that proportion rising to 80% by the time they are six years old**.However, when food allergies are complicated by other allergic conditions, **tolerance** becomes more difficult to acquire.

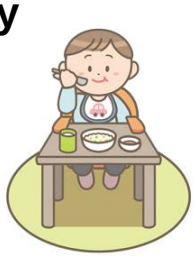

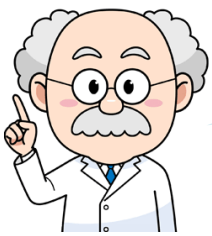

Tolerance is **when allergic symptoms do not occur, even if the person afflicted with an allergy consumes as much of the allergenic food as they want, whenever they want**.
We consider treatment methods for food allergies by distinguishing **a situation in which the allergenic food can be eaten on a one-time basis**.

What kinds of things are challenging about food allergies?

I always have to keep an eye on my child...

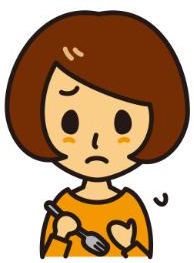

Parents experience pressure and stress in the need to prepare meals that don't contain the allergenic food and making sure that the child doesn't have allergic symptoms, and the concern that the child may accidentally consume the allergenic food.

I'm different from my friends...

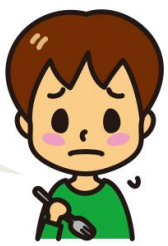

Some children develop a fear of a particular food after experiencing an allergic reaction. Others have painful experiences because they cannot eat the same things as their friends during their time at school or kindergarten.

What kinds of treatments for food allergies are there?

The main treatments for food allergies currently being undertaken in Japan are **elimination diet therapy** and **oral immunotherapy**, the latter of which is still in the research stages. First, let's learn about the characteristics of each of these treatments.

Characteristics

Elimination diet therapy

1) At present, this **treatment method is available on a general basis** for food allergies.

2) This treatment involves eliminating a food that triggers symptoms when eaten during everyday life, Based on guidance from a physician.

3) There are cases in which it is possible to eat condiments and so on that contain the allergenic food in small amounts, so the scope of the elimination diet is decided based on consultation with a physician.

4) In some cases, a child **naturally acquires a tolerance as they grow up and becomes able to eat the allergenic food.**

Oral immunotherapy

1) At present, this treatment method is in the research stages, so it is not available **on a general basis**.

2) Based on guidance from a physician, this treatment method involves **actively consuming the allergenic food** during everyday life.

3) Criteria for the amount and frequency of eating the allergenic food are not fixed, **so decisions are tailored to each individual child.**

4) **One of the goals of this treatment is to generate a tolerance to consuming and coming into contact with an unexpected allergenic food**.

5) At present, this treatment method **does not completely cure food allergies and does not enable the patient to eat the allergen whenever they want.**

What does each treatment involve?

On page 7, we told you about the characteristics of elimination diet therapy and oral immunotherapy. Let's find out about what each of these therapies involve.

Elimination diet therapy

What is elimination diet therapy?

As the name suggests, this treatment method involves your child avoiding the food that causes allergic symptoms while partaking in their usual eating habits.

Sometimes it is possible to use this method while adding small amounts of the allergen to meals as a condiment or seasoning, so the extent of elimination is decided based on consultation with your physician. If, for example, eliminating the allergen means that your child is not consuming enough protein, it may be necessary to supplement their diet with an alternative foodstuff.

What procedure does it involve?

In order to discuss how things are progressing at home and to assess the nutritional balance of your child's diet between medical examinations, you will need to go to the hospital on a regular basis. There, you will be asked if you have been able to maintain the elimination diet at home, whether your child has experienced any allergic reactions, and if necessary, to undergo blood tests or sensitivity tests.* Your physician will then decide how to continue the treatment or whether to make any changes.

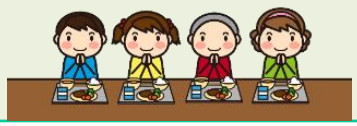

*Sensitivity test: This is a test in which your child consumes the food that causes allergic symptoms to see whether those symptoms actually occur. It is undertaken in order to check whether they can now consume the allergenic food having eliminated it from their diet (whether they have been able to acquire a tolerance). Depending on your child's condition, they undergo the test as an outpatient or an inpatient.

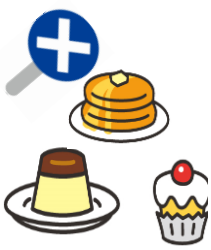

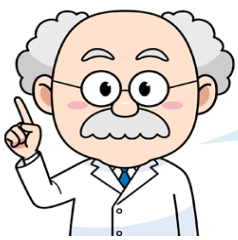

If your physician decides it is unlikely that your child will naturally become able to consume an allergenic food even though they have reached a certain age, the physician may recommend some kind of immunotherapy.

Oral immunotherapy

What kind of treatment method is oral immunotherapy?

First, the amount of the food to be eaten is determined based on a sensitivity test. While monitoring any allergic reactions, this amount is gradually increased, and at the point when it becomes possible to consume the target amount, your child continues to eat that amount for a certain period of time.

What procedure does it involve?

Example

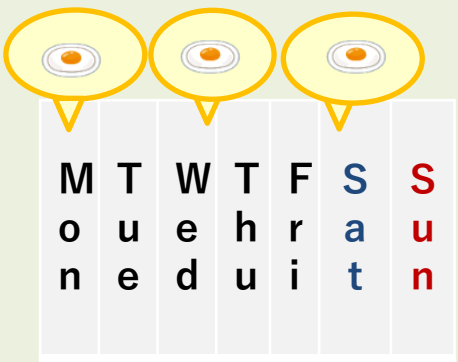

■ Your child consumes the allergenic food at home on the days and in the amounts decided by their physician. In some cases, they will consume the food every day.

■ Having consumed the allergenic food, your child is monitored and if any allergic symptoms are observed, they take prescription medication. If the symptoms are severe, you may have to call an ambulance or be examined as an outpatient.

■ Oral immunotherapy is a treatment method in which the aim is for your child to **acquire a tolerance** by deliberately consuming the allergenic food. At present, this treatment method is not available on a general basis in Japan and is regarded as being **in the research stages**.

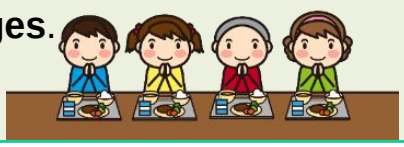

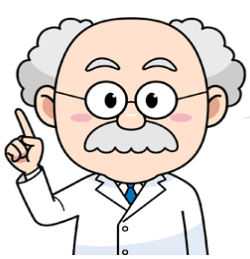

The amount of a food to be consumed at any one time, the extent to which that amount is increased, and so on are determined by a physician in line with your child's condition.

Depending on the amount consumed and the severity of the symptoms, sometimes oral immunotherapy cannot be undertaken.

Step 3

Understanding the lifestyle and psychological impact of treatments

In Step 3, let's look at how elimination diet therapy and oral immunotherapy affect children and their parents and guardians from six different perspectives.

How does each treatment affect the everyday lives of you and your child?

Due to the act of eating, which is essential for human survival, a food allergy causes allergic symptoms and can sometimes induce life-threatening illnesses. As such, it is known as a condition that has a significant impact on people's **quality of life**.

Quality of Life

① **Effects**

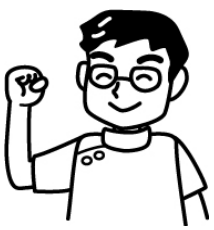

② **Side-effects**

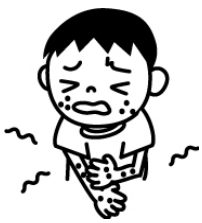

③ **Treatment period**

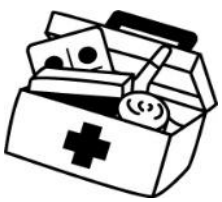

④ **Cost**

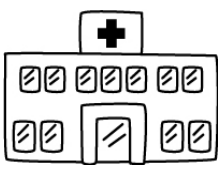

⑤ **Psychological aspects**

Impact on the child

Impact on parents and gu

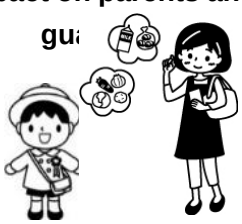

⑥ **Lifestyle**

Impact on the child

Impact on parents and guardians

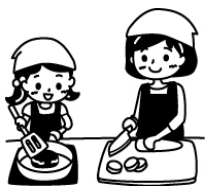

Step 3

Understanding the lifestyle and psychological impact of treatments

① Effects

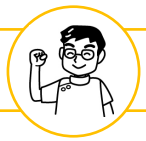

Elimination diet therapy

As your child grows up, they may naturally become able to eat the allergenic food. For example, it has been reported that **about 50% of children with an egg allergy naturally become able to eat it by the time they are four and a half years old, while that ratio rises to about 80% by the time they are six years old**.

Oral immunotherapy

There is still **insufficient** evidence to demonstrate specific ratios, but for example, it has been shown that with oral immunotherapy for egg allergies, **there is a strong possibility that tolerance can be acquired** more quickly than with elimination diet therapy. However, symptoms sometimes reappear if your child consumes the allergenic food after a period of time during which they stopped doing so, so it is necessary to follow the instructions of your physician.

② Side-effects

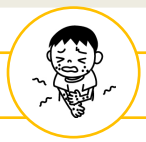

Elimination diet therapy

There are no adverse reactions to the act of eliminating the allergenic food itself. But if there are concerns that eliminating the allergen has led to your child no longer consuming enough protein, it may be necessary to supplement their diet with an alternative foodstuff. Because there is always a risk of accidentally ingesting the food allergen, children who may be prone to anaphylactic shock also need to carry an **EpiPen.***

Oral immunotherapy

Compared with elimination diet therapy, there is thought to be a strong possibility that itchiness in the mouth, discomfort, stomachache, and other such symptoms will occur as a result of consuming the allergenic food. There are sometimes cases of anaphylactic shock, which is the most critical of these symptoms. For this reason, it may be necessary for your child to carry an **EpiPen*** with them at all times.

[*What is an EpiPen?]

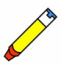

An EpiPen is used when a person exhibits anaphylactic symptoms. It temporarily alleviates these symptoms and is a supplementary treatment to prevent anaphylactic shock, so consultation and treatment by a physician must immediately be sought after injection with an EpiPen.

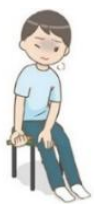

Step 3

Understanding the lifestyle and psychological impact of treatments

③ Treatment period

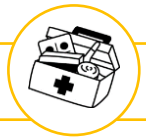

Elimination diet therapy

As a rule, elimination of the allergenic food from your child's diet will continue until their allergic symptoms no longer occur. There is no way of knowing how long that will take, so the treatment may continue for a long time.
During that time, it will also be necessary to regularly go to hospital and to consult with your physician as to whether the elimination diet therapy should continue.

Oral immunotherapy

Some children only need to undergo the treatment for six months, while for others, it will continue for several years.
If treatment is suspended partway through, your child's inability to consume the allergenic food may reoccur, so long-term consumption of the allergenic food is required in order to maintain a state in which there is no allergic reaction.

④ Cost

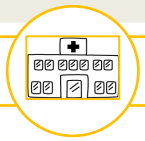

Elimination diet therapy

It is necessary to continue going to hospital **about once every three to six months**, and where appropriate, your child may undergo sensitivity tests to assess whether allergic reactions occur.
If your child is not consuming enough protein, you may also need to purchase alternative foods (substitute foods), and all these things can give rise to additional costs.

Oral immunotherapy

Your child needs to go to hospital **about once a month** to monitor their condition, so hospital visits are more frequent than with elimination diet therapy and can give rise to additional costs.
The same applies to sensitivity tests for assessing the amount of the allergenic food that your child can consume, and these tests may necessitate their hospitalization.

Step 3

Understanding the lifestyle and psychological impact of treatments

⑤ Psychological aspects

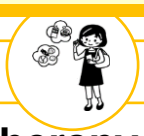

Elimination diet therapy

Parents

Having to make elimination-based foods for every meal and the fact that dining out presents difficulties can feel burdensome for parents. You may also feel anxious and stressed about always having to check that your child does not accidentally ingest or come into contact with the allergenic food.

Children

Depending on how old your child is, they may feel stressed about eating different foods from those around them, or worry that their symptoms may be triggered by accidentally ingesting the allergenic food. However, children who have grown accustomed to elimination diet therapy are unlikely to report feeling stressed about the elimination itself.

Oral immunotherapy

Parents

Preparing meals that adhere to the amounts indicated by your physician and continuing to ensure that your child eats those meals at home can put a strain on you as a parent. During mealtimes, you can also feel anxious or stressed about the need to check if your child is exhibiting allergic symptoms.

Children

Children sometimes refuse to eat a food if they dislike it or it feels unpleasant in some way. Treatment is sometimes suspended if your child feels particularly averse to consuming that food. However, if the extent to which they can consume a food—even in small amounts— increases, this can motivate them to persevere.

⑥ Lifestyle

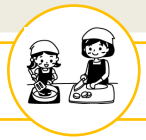

Elimination diet therapy

Parents

You need to inform kindergartens, nurseries, and schools of the fact that your child is undergoing elimination diet therapy and how to respond if they accidentally ingest the allergenic food.

Children

If your child accidentally eats the food allergen or if they experience allergic symptoms, they need to ask somebody for help.

Oral immunotherapy

Parents

You need to inform kindergartens, nurseries, and schools of the fact that your child is undergoing treatment for a food allergy and how to respond if they exhibit allergic symptoms.

Children

Your child needs to understand that they are undergoing treatment, and to ask somebody for help if they experience allergic symptoms.

[Quality of life]
In research to date, it is reported that quality of life improves for the parents and guardians of children undergoing oral immunotherapy. However, it is not yet known if the same applies to the children themselves, about whom little has been reported.

Step 3

Understanding the lifestyle and psychological impact of treatments

So far, we have told you about the characteristics of each treatment method from a medical point of view, and from the point of view of the psychology and lifestyles of children, parents, and guardians. This is by no means all the information you will need, so if there is anything you do not understand, that you are worried about, or that you would like to check, please make a note of it here and ask about it at your child's next hospital visit.

MEMO

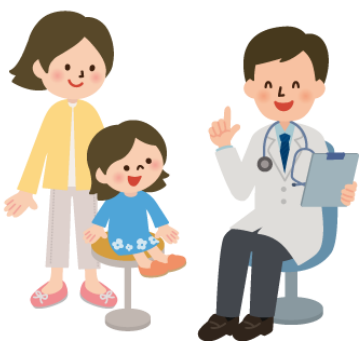

Step 4

Clarifying what is important to you when you make a decision

In **Step 4**, let's consider what is important to you when you choose a treatment.
Each treatment method has its advantages and disadvantages.
By considering what is important to you based on the information we have provided so far, you should be able to make a more informed decision.

But even if someone asks, "What is important to you?" it is hard to come up with an answer straight away, isn't it?
That is why we have compiled the following questions, which we hope you will find helpful.
Parents and guardians should make final decisions taking into consideration their child's age and level of development, and by listening to the opinion of their child, **who is the one undergoing treatment.**

How your child feels about treatment

Have you ever heard of the United Nations Convention on the Rights of the Child?
As the name suggests, the Convention on the Rights of the Child was created to protect children's rights, and one of the articles it contains states that **"Children have the right to give their opinions freely on issues that affect them."**
This was established with the goal that everyone can have an awareness of the need to respect children's feelings and opinions.
Parents cherish and want to protect their child, and when they are making various decisions, sometimes the child may be unable to convey how they themselves feel.
We hope that you will take this opportunity to speak with your child, to enable them to notice their own feelings, and to nurture their ability to convey those feelings to others.

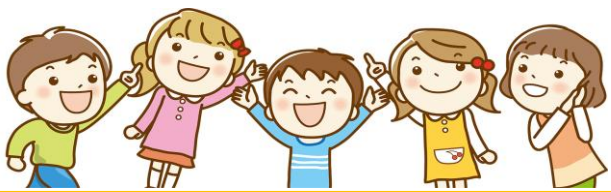

Step 4

Clarifying what is important to you when you make a decision

This page is for making notes that will enable you to understand your child's feelings in relation to their condition and its treatment. We have provided some questions that we hope you will find helpful.

MEMO

What understanding does your child have of their condition?

How does your child feel about the treatment of their food allergy?

Other feelings your child has about their condition and its treatment.

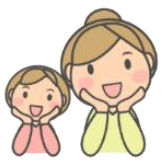

Step 4

Clarifying what is important to you when you make a decision

Let's answer some questions

The final decision about what treatment method to choose is made based on cooperation between parents and guardians and their physician, but it is natural to feel hesitant at this point. With this in mind, in order for you to **organize your thoughts** about **what is important to you when making this decision**, try answering the following questions.

Please answer each question by describing how you feel at this moment in time. Circle the number that you think most closely matches your feelings. To help you make a decision, the area you circled indicates which treatment method you are inclined to in relation to the question asked.

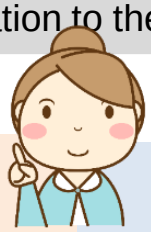

Elimination diet therapy

Oral immunotherapy

◇ I do not yet have as much evidence as I would like, but in relation to unexpected ingestion of the allergenic food, my expectations for the possibility that my child's condition will reach a safe level are:

	1	2	3	4	5	
Not important						Important

◇ There is a possibility that ingesting the allergenic food will cause allergic symptoms or an anaphylactic reaction.

	1	2	3	4	5	
Cannot accept						Can accept

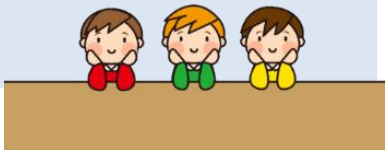

Step 4

Clarifying what is important to you when you make a decision

What did you think about your answers to the questions on page 16? In the same way, circle the number that you think most closely matches your current feelings in relation to the following questions. Please refer to your answers when you make a decision regarding treatment.

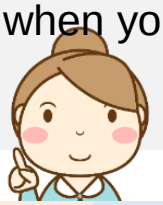

Elimination diet therapy	Oral immunotherapy
Preparing therapeutic meals (the allergenic food) and managing allergic symptoms is difficult.	I can prepare therapeutic meals (the allergenic food) and manage allergic symptoms.
1 2 3 4 5	
It is best that my child goes to hospital as little as possible.	I do not mind if my child goes to hospital fairly often.
1 2 3 4 5	
My child does not want to undergo oral immunotherapy.	My child feels like they can undergo oral immunotherapy.
1 2 3 4 5	

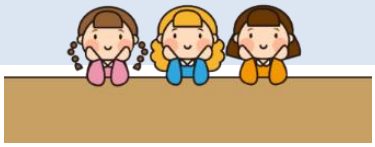

Step 5 Clarifying your current feelings and organizing your concerns

In **Step 5**, let's clarify your thoughts about choosing a treatment at this moment in time.

While referring to the results of your answers to the questions on pages 17 and 18, check the boxes below in relation to your thoughts **right now** about treatment methods for food allergies.

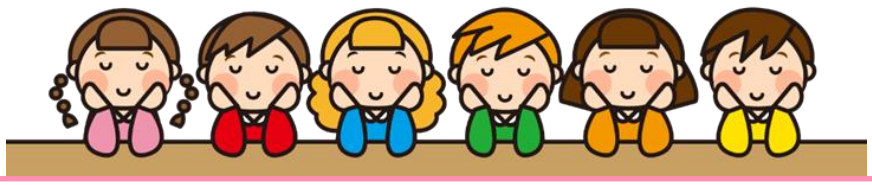

☐ We will try elimination diet therapy

☐ We will try oral immunotherapy

☐ We have not yet been able to decide

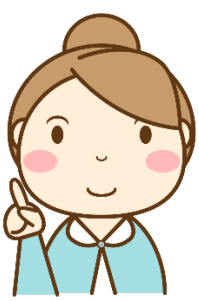

As we have said, your choice right now does not have to be definitive. You can change your mind at any time, so if there is anything you are uncertain or worried about, feel free to discuss it with your attending physician or with healthcare staff.

Step 5

Clarifying your current feelings and organizing your concerns

So far, you have been provided with information about treatment methods and organized your thoughts. This will be the final set of questions.

The following questions are for understanding the extent to which parents and guardians are ready to decide the food allergy treatment method for their child. Please check the boxes ☑ for “Yes” or “No” for the answers that apply to you.

**Do you think** that your choice is the best one for you and your child?	Yes ☐ No ☐
Do you understand the advantages and disadvantages **of the options available?**	Yes ☐ No ☐
Are you clear about which advantages and disadvantages are the most important for you?	Yes ☐ No ☐
Are you receiving enough support and advice to make your choice?	Yes ☐ No ☐

The SURE Test © O’Connor and Légaré, 2008 (Japanese translation)
Partly adapted from Osaka et al., 2019 (Japanese translation)

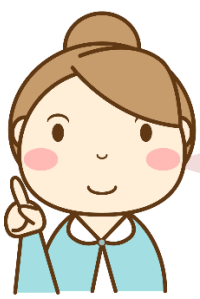

What were your answers?
If you answered “No” to one or more of the questions, there may be aspects of the treatments that you do not yet fully understand, or perhaps you are hesitant about your decision. If that is the case, try re-reading Steps 1 to 4 and organize your thoughts little by little, for example, by consulting your physician or healthcare staff the next time your child has a medical examination.

Step 5

Clarifying your current feelings and organizing your concerns

We believe that by sufficiently considering the impact on you and your child and reaching a decision that you find acceptable, you will be able to abide by the conditions to which you have agreed as the treatment progresses. If you still have any concerns, make a note of them here.

MEMO

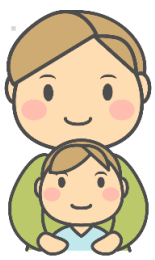

MEMO

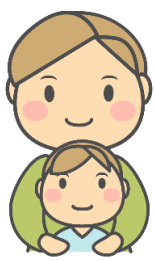

Conclusion

While reading through this guidebook, were you able to consolidate your knowledge and your thoughts or make a decision in line with your wishes? Choosing a treatment method is very difficult, isn't it? Even if you make a decision, you can change your mind at any time, so going forward, if you have any doubts about your choice of treatment method, do please talk with your clinician or healthcare staff.

Whichever choice you make, the most important thing is that you continue to be open in dealing with your child's food allergy.
We hope that parents and children can be satisfied with the treatment being undertaken, and that you will be able to confront the condition together with all healthcare staff involved, including your physician.
Then, one day in the future, we sincerely hope that your child will recover from their food allergy.

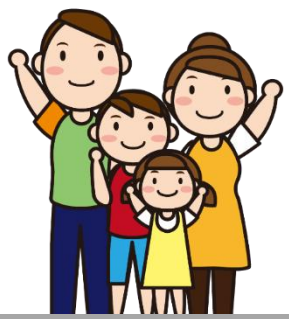

This guidebook has been produced based on information correct as of July 2020.
The treatment of food allergies is a field in which research is still being advanced, and we will continue to investigate and reexamine such information going forward.
When you use this guidebook, please check the dates on which information has been updated.

About the development of this guidebook

Among others, this guidebook was produced based on the views of parents and guardians whose children have food allergies, medical specialists in pediatric allergies, nurses, pediatric care researchers, and decision-making researchers. Furthermore, the medical information contained in the guidebook has been subject to supervision by pediatric allergy specialists responsible for the treatment of food allergies.

This guidebook was also produced with the assistance of Grants-in-Aid for Scientific Research (KAKENHI), Aside from this, the guidebook has not received any corporate funding assistance (i.e. there has been no conflict of interest).

[Sources/references]

1. B Duca, N Patel et al., *GRADE-ing the Benefit/Risk Equation in Food Immunotherapy*, Current Allergy and Asthma Reports, Volume 19, article number 30, 2019
2. O Romantsik, M Tosca, S Zappettini et al., *Oral and sublingual immunotherapy for egg allergy*, Cochrane Database of Systematic Reviews, Issue 4, 2018
3. *Japanese Pediatric Guideline for Food Allergy*, Food Allergy Committee, Japanese Society of Pediatric Allergy and Clinical Immunology, 2021
4. *User-friendly Guide to Food Allergy Measures for Asthma Prevention*, Environmental Restoration and Conservation Agency, 2014
5. *Food Allergy Handbook 2014: For those Involved in Children's Diets*, Japanese Society of Pediatric Allergy and Clinical Immunology
6. P Bégin, ES Chan, H Kim et al., *CSACI guidelines for the ethical, evidence-based and patient-oriented clinical practice of oral immunotherapy in IgE-mediated food allergy*, Current Allergy and Asthma Reports, Volume 16, article number 20, 2020
7. Edited by K Nakayama, T Iwamoto, *Patient-focused decision-making support: Care for acceptable decisions*, Chuohoki Publishing Co., Ltd., 2012
8. Edited by T Nakayama, *Starting now! Shared decision-making: New healthcare communication*, Japan Medical Journal, 2017
9. M Greenhawt, M Shaker, T Winders et al., *Development and acceptability of a shared decision-making tool for commercial peanut allergy therapies*, Ann Allergy Asthma Immunol, Volume 125, Issue 1, pp. 90–96, 2020
10. Supervising editor M Ebisawa, *Confidently answering questions from parents: Pediatric allergy Q&A*, Japan Medical Journal
11. *Human Rights of the Children*, Ministry of Foreign Affairs
https://www.mofa.go.jp/policy/human/child/index.html

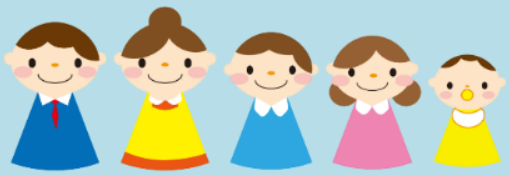

Let's Think Together. Treatment of Food Allergies

—To all those who are considering treatment options—

[Developers]

Tokai University School of Medicine Faculty of Nursing, Junko Hayama
St. Luke's International University Kazuhiro Nakayama

[Medical supervision]

Tokai University School of Medicine, Hachioji Hospital Koichi Yamaguchi
Tokai University School of Medicine, Hachioji Hospital Kota Hirai
Tokai University School of Medicine, Department of Pediatrics Hiroyuki Mochizuki
