# Supplementary material for: Developing a Parent-Focused Decision Aid to Promote Child-Inclusive Shared Decision-Making in Pediatric Oral Immunotherapy: Pragmatic Exploratory Feasibility Study
Source: J Particip Med. 2026 Jan 6;18:e77782. doi: 10.2196/77782 (PMC12774402; doi:10.2196/77782)
Supplement: Multimedia Appendix 2 [file jopm-v18-e77782-s002.docx]

**Semi-structured interview guides (parent–child).**

These guides provide prompts for semi-structured interviews. Wording may be adapted to the participant’s age and context. DA = decision aid.

**Interview Guide (Parents)**

・Since receiving the DA, how have you considered the treatment options? Please describe the process, including the respective roles of the mother and father.

・When deciding on your child’s treatment, with whom did you mainly consult? (e.g., spouse, your own parents, the child, etc.)

・At this point, what choice have you made regarding your child’s treatment?

・What did you explain to your child about making the treatment decision, and what conversations did you have? How did your child respond?

・What are your views on your child’s involvement in making the treatment decision?

・How did you use the decision aid?

・When using the decision aid to choose a treatment, what aspects did you find helpful, and what aspects did you find difficult?

**Interview Guide (Children, ages 6–9)**

・When you learned about ways to help your food allergy, who did you talk to? What things were easy to understand? What was hard to understand?

・When you talk about how to help your food allergy, who do you talk with? (For example, your mom or dad, your teacher, or your doctor.)

・After hearing about the ways to help your food allergy, how do you feel? For example, do you think “I should not eat foods that make me sick,” or “I want to try the treatment where I eat a little,” or “I am not sure yet”? Please tell me if you want to try or do not want to try something.

・How would you like decisions to be made about how to help your food allergy? (For example, “I want my family to decide,” “I want to decide together,” or “I want the doctor to decide.”) Please tell me what you would like.

**Interview Guide (Children, ages 10–16)**

・About treatment for your food allergy, who have you talked with? What was clear to you? What was hard to understand?

・When talking about what to do next for treatment, who do you talk with? (For example, your parent(s), teacher, or doctor.)

・After hearing about the treatment choices, how do you feel? For example, do you think “I should avoid the foods,” “I want to try the treatment where I eat small amounts,” or “I am not sure”? If you can, please tell us why you feel that way.

・How would you like decisions to be made about your treatment? For example, “I want my family to decide,” “I want to decide myself,” “I want the doctor to decide,” or “I want

to decide together.” Please tell us what you prefer.
