# Supplementary material for: Developing a Parent-Focused Decision Aid to Promote Child-Inclusive Shared Decision-Making in Pediatric Oral Immunotherapy: Pragmatic Exploratory Feasibility Study
Source: J Particip Med. 2026 Jan 6;18:e77782. doi: 10.2196/77782 (PMC12774402; doi:10.2196/77782)
Supplement: Multimedia Appendix 3 [file jopm-v18-e77782-s003.docx]

De-identified parent–child excerpts by dyads A–D.

This appendix presents representative, de-identified excerpts from semi-structured interviews with parents and children. Dyads are labeled consistently (e.g., Parent A / Child A). Full transcripts are not included to protect confidentiality.

**Dyad A (age 7, elimination preference)**

Parent A: We thought elimination was manageable, and since my child had never eaten XX, it didn’t feel like a major loss. Trying OIT seemed too risky and burdensome.

Parent A: If I said, “let’s try this,” my child would refuse because of the painful memory from the last reaction.

Child A: My mother decides… I don’t know.

Child A: I’m not really sure what OIT is.

**Dyad B (age 10, emerging interest)**

Parent B: At first, I didn’t think much about OIT. But when I asked directly, I was surprised that my child had such clear thoughts.

Parent B: It made me realize that I should ask my child first before making any decisions.

Child B: I want to talk more about the treatment… I’ve been kind of thinking about it.

Child B: I’d been thinking, “I want to say something,” but kept it to myself.

**Dyad C (age 14, Favoring OIT)**

Parent C: It should be the child’s choice, since it is their body and burden.

Parent C: If OIT could reduce the constant anxiety, I would fully support it.

Child C: I read the DA with my parent… I’d like to try OIT and have a say when deciding.

Child C: How can I eat without worrying about symptoms? That’s what I’ve been thinking about.

**Dyad D (age 14, elimination preference)**

Parent D: Since reactions were severe, elimination felt safer. I wasn’t very familiar with other treatment options.

Parent D: Even after reading the DA, I felt it would be better to ask the doctor directly.

Child D: I’m fine with how things are, and I don’t really feel the need to talk about treatment.

Child D: I think I’ll just live with it; I don’t expect much to change.
